# Supplementary figures and images for: Genome-wide analyses reveal the contribution of somatic variants to the immune landscape of multiple cancer types
Source: PLoS Genet. 2024 Jan 19;20(1):e1011134. doi: 10.1371/journal.pgen.1011134 (PMC10829993; doi:10.1371/journal.pgen.1011134)

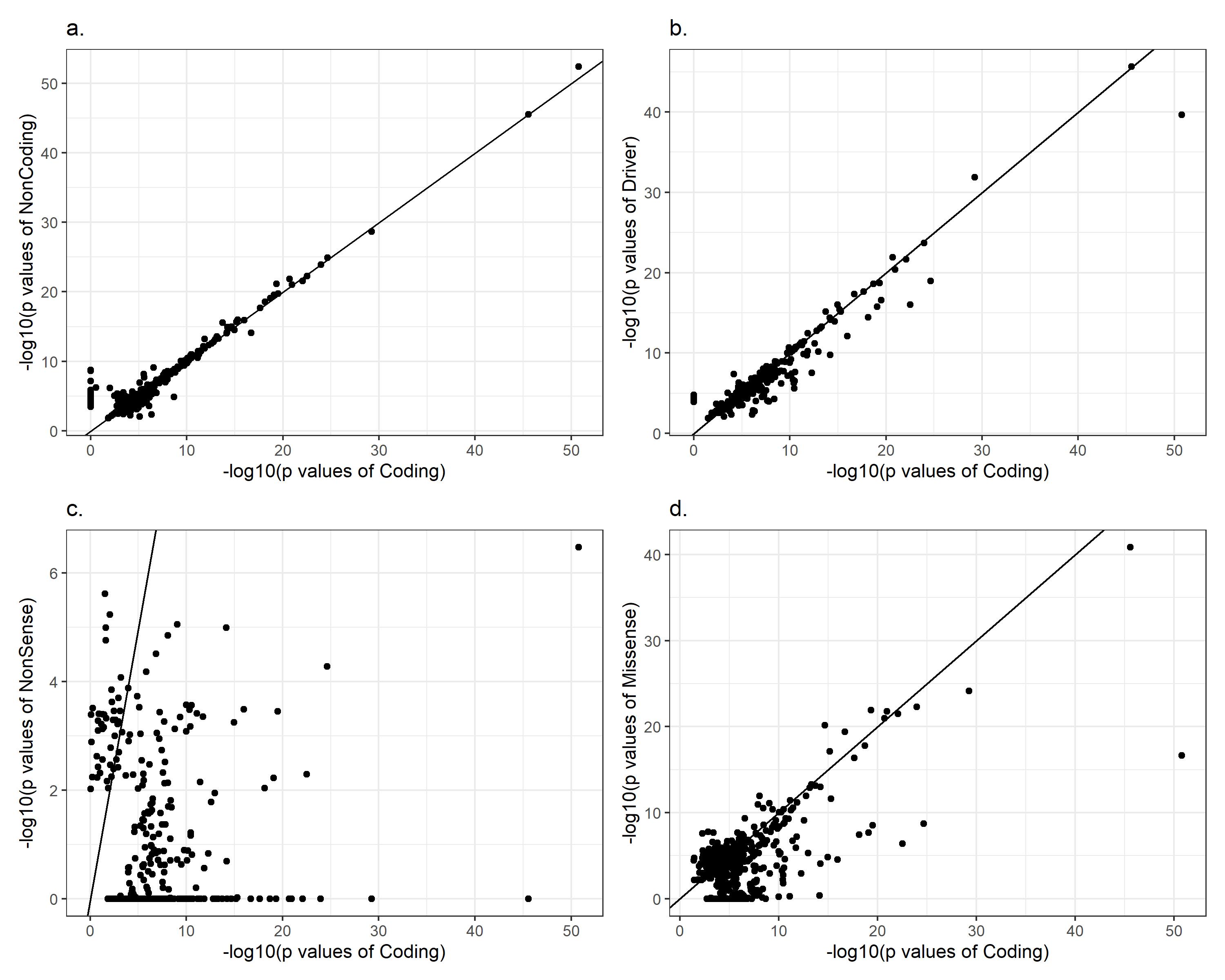

Supplement: S1 Fig — In all the four panels, the x-axis corresponds to the analysis including only coding regions (denoted as Coding). The y-axis corresponds to the alternative analyses as below. (a) the somatic mutations in non-coding regions were additionally included (denoted as NonCoding); (b) driver somatic mutations were double-weighted (denoted as Driver); (c) only somatic mutations classified as Nonsense Mutation were included (denoted as Nonsense); (d) only somatic mutations classified as Missense Mutation were included (denoted as Missense). In our analysis, genes with fewer than 5 somatic mutation carriers were excluded for both coding and alternative analyses. Consequently, the sets of genes included in different analyses were not identical. For genes not included in either coding or alternative analyses, we set the corresponding p-value to 1. (JPEG) [file pgen.1011134.s011.jpeg]
