# Supplementary material for: Genome-wide analyses reveal the contribution of somatic variants to the immune landscape of multiple cancer types
Source: PLoS Genet. 2024 Jan 19;20(1):e1011134. doi: 10.1371/journal.pgen.1011134 (PMC10829993; doi:10.1371/journal.pgen.1011134)
Supplement: Attachment — Submitted filename: Response_to_Reviewers_2023-12-12.docx [file pgen.1011134.s012.docx]

We would like to thank both the reviewers for their careful examination of the manuscript and positive feedback. They raised several important points and we have revised the manuscript accordingly. Please find below the comments from the reviewers (in italics) and our responses after each comment. To assist reviewers and editors in pinpointing the modified sections, line numbers have been incorporated into the ***revised manuscript***.

**Reviewer #1:** *In this study Bi et al. taking advantage of TCGA dataset to analyze the genome-wide association between genetic mutations and immune traits in the tumor microenvironment in different cancer types. They found that distinct gene mutations account for immune traits in different cancer types. In the end, they focused on the KEAP1 gene. The mutation of which is associated with NRF2 pathway activation, and consequently remodel the tumor immune microenvironment in lung adenocarcinoma. More importantly, the NRF2 activation status could serve as a better responsive biomarker for immune therapy. This is a very interesting study, and may have translational values as well.*

**We would like to thank you for the positive comments.**

*Here are a few minor concerns that can be addressed*

*1.      All the analysis and conclusions are drawn from bioinformatic analysis. It would be great to validate some key findings experimental. This is minor, as it would be very challenging to get patient samples to confirm gene mutation or signal pathway activation.*

**Response:** We acknowledge the pivotal role of experiments in validating our findings. Unfortunately, due to the time constraint for the revision and other issues highlighted by the reviewer, conducting relevant experimental work is challenging for this research. Demonstrated as below, we have incorporated relevant statements in the Discussion Section.

**(lines 381-383)**	“Please note that all conclusions have been drawn from bioinformatic analyses. We fully acknowledge the importance of experimental validation as a necessary step for future research.”

*2.      The study mainly focused on somatic mutations in coding regions. As non-coding regions (Long non-coding RNAs) also greatly contribute to TME remodeling. Would it be possible to include them as well?*

**Response:** Thank you for the insightful comment. In the initial submission, we focused on coding regions due to their predictable functional impact. In the revised version, we have expanded our scope by conducting additional genome-wide analyses to include somatic mutations in non-coding regions as well. The results are highly consistent to the results in the initial submission (see Figure S1 in the end of the response letter). We have also incorporated relevant statements in the Discussion Section.

**(lines 367-375)**	“In the main text, the genome-wide analyses mainly focused on coding regions and treated the somatic mutations equally. We noticed the research studies that highlighted the important role of noncoding RNAs in remodeling TME.^49,50^ Hence, we also conducted multiple sensitivity analyses in which 1) somatic mutations in non-coding regions were included; 2) only somatic mutations classified as missense or nonsense were included; and 3) driver somatic mutations were double-weighted. The definition of the driver somatic mutation is from OncoVar^51^, an integrated database and analysis platform for oncogenic driver variants in cancers. The detailed results can be found in Figure S1 and Tables S6-S9. In general, additionally including somatic mutations in non-coding regions or upweighting driver mutations does not significantly help find more findings.”

**Reference:**

Wang, T., Ruan, S., Zhao, X., Shi, X., Teng, H., Zhong, J., You, M., Xia, K., Sun, Z., and Mao, F. (2021). OncoVar: an integrated database and analysis platform for oncogenic driver variants in cancers. Nucleic acids research 49, D1289-d1301. 10.1093/nar/gkaa1033.

Yang, J., Xu, J., Wang, W., Zhang, B., Yu, X., and Shi, S. (2023). Epigenetic regulation in the tumor microenvironment: molecular mechanisms and therapeutic targets. Signal Transduction and Targeted Therapy 8, 210. 10.1038/s41392-023-01480-x.

Lv, Y., Lv, Y., Wang, Z., Yuan, K., and Zeng, Y. (2022). Noncoding RNAs as sensors of tumor microenvironmental stress. Journal of Experimental & Clinical Cancer Research 41, 224. 10.1186/s13046-022-02433-y.

*3.      The authors investigated the associations between mutations and TME traits. Would it be possible to classify types of gene mutations and TME traits? Such as deletion, nonsense mutation or missense mutation.*

**Response:** Thanks for the suggestion. We summarized the counts of different somatic mutation types in Table S10. The most commonly observed somatic mutation type is Missense mutation (~52.9%). In addition, Nonsense mutation takes around 4% and other types of protein altering mutations, such as deletion, do not take more than 3%. Thus, we conducted two additional genome-wide analyses in which only nonsense mutation or missense mutation was included (see Figure S1 in the end of the response letter). If the analysis targeted Missense Mutation only, a significant majority of the initially identified signals were successfully replicated. Meanwhile, when the analysis confined to Nonsense Mutations exclusively, only a limited number of the initially identified signals were reproduced. More details can be found in the below.

**(lines 376-381)**	“If the analysis targeted Missense Mutation only, a significant majority of the initially identified signals were successfully replicated. That is because Missense Mutation takes more than 52.9% of the somatic mutations (Table S10). Meanwhile, when the analysis confined to Nonsense Mutations exclusively, only a limited number of the initially identified signals were reproduced because Nonsense Mutation only takes ~ 4% of the somatic mutations (Table S10). And both analyses do not exclusively identify many signals missed by the initial analyses.”

**Reviewer #2:** *The authors performed genome-wide analysis and found associations between somatic mutations and immune landscape in various cancer types. In the case study of lung adenocarcinoma, the authors demonstrate KEAP1 mutations activate the NRF2 pathway and further lead to decreased T cell infiltration and increase T cell exhaustion. They also curated a NRF2 gene signature that shows better patient stratification in anti-PD1 therapy response than CD274 (PD-L1) alone. Overall, the study is clear and has made interesting findings, but definitely needs more work on emphasizing novelty and significance, rationalizing the study design, and polishing the figures (See details below).*

**We would like to thank you for the positive comments.**

*Major comments:*

*1.      KEAP1 and NRF2 have been studied in NSCLC as well as other cancer types. It is not clear what discoveries have been made prior to this work, and how this work builds upon and extends beyond previous findings. Providing such context in the introduction helps establish the significance of the study.*

**Response:** Thanks for the suggestion. We agree that the introduction of the previous findings can help clarify the significance of our research. In Introduction Section, we gave a brief relevant introduction as below.

**(lines 110-118)**	“Comprehensive genomic analyses have identified somatic mutations and other alterations in the *KEAP1* or *NRF2* genes in various types of cancer, and Nrf2 mutations occur less frequently than Keap1 mutations.^19^ Disruptions in the Keap1-Nrf2 pathway is frequently associated with poor prognosis and chemotherapeutic resistance in NSCLC.^20^ Since the Keap1-Nrf2 pathway plays as primary regulator of key cellular processes that contribute to resistance against chemotherapy drugs, NRF2 has been studied as a potential therapeutic target molecule in NSCLC and some other cancers.^21^ Our findings explore the molecular features and the impact of KEAP1-NRF2 to TME, which will be beneficial for novel treatment approaches in NSCLC in the near future.”

**Reference**

Taguchi, K., and Yamamoto, M. (2017). The KEAP1-NRF2 System in Cancer. Front Oncol 7, 85. 10.3389/fonc.2017.00085.

Singh, A., Misra, V., Thimmulappa, R.K., Lee, H., Ames, S., Hoque, M.O., Herman, J.G., Baylin, S.B., Sidransky, D., Gabrielson, E., et al. (2006). Dysfunctional KEAP1-NRF2 interaction in non-small-cell lung cancer. PLoS Med 3, e420. 10.1371/journal.pmed.0030420.

Cuadrado, A., Rojo, A.I., Wells, G., Hayes, J.D., Cousin, S.P., Rumsey, W.L., Attucks, O.C., Franklin, S., Levonen, A.L., Kensler, T.W., and Dinkova-Kostova, A.T. (2019). Therapeutic targeting of the NRF2 and KEAP1 partnership in chronic diseases. Nat Rev Drug Discov 18, 295-317. 10.1038/s41573-018-0008-x

*2.      The impact of findings in the discussion needs to be strengthened. For example, authors highlighted that the curated NRF2 signature can serve as a better biomarker than PD-L1, but lacking the discussion about how this biomarker can be used practically in the clinical setting, considering RNA-seq is not routinely performed as a screening method.*

**Response:** We appreciate the reviewer’s great comments. Figure 4b shows that KEAP1 mutation is the major driver for NRF2 pathway activation in LUAD patients. In clinical setting, KEAP1 mutation test is a more applicable test than RNA-seq to check NRF2 signature gene expression. We recommend using KEAP1 gene sequencing as a companion test in clinical trials to select patient for anti-PD1 or anti-PD-L1 therapy. We have added the recommendation for clinical useage of our discovery in manuscript acoordingly.

**(lines 320-324)**	“However, sequencing a single gene is much easier than doing expression quantification of a panel genes. Since KEAP1 mutations cause upregulation of NFR2 and its downstream signature genes which is confirmed in our analysis (Figure 4b), KEAP1 gene sequencing can be a more applicable test to be used in clinical setting than NFR2 gene signature quantification by either RNA-seq or microarray.”

*3.      Are known driver genes and passenger genes treated differently in this study? Elaborate how the current method allows discovery of causal relationship between somatic mutations and immune landscape in TME. If not, provide discussion about the limitation of the method design.*

**Response:** In the initial submission, we conducted genome-wide analyses without prior information of driver gene/mutation and passenger gene/mutation. We agree that incorporating information of driver mutation can help identify true causal relationship, rather than association relationship. In the revised version, we downloaded driver mutation information from OncoVar Dataset and re-conducted genome-wide analysis to highlight the driver mutation.

**(lines 367-375)**	“In the main text, the genome-wide analyses mainly focused on coding regions and treated the somatic mutations equally. We noticed the research studies that highlighted the important role of noncoding RNAs in remodeling TME.^49,50^ Hence, we also conducted multiple sentitivity analyses in which 1) somatic mutations in non-coding regions were included; 2) only somatic mutations classified as missense or nonsense were included; and 3) driver somatic mutations were double-weighted. The definition of the driver somatic mutation is from OncoVar^51^, an integrated database and analysis platform for oncogenic driver variants in cancers. The detailed results can be found in Figure S1 and Tables S6-S9. In general, additionally including somatic mutations in non-coding regions or upweighting driver mutations does not significantly help find more findings.”

**Reference:**

Wang, T., Ruan, S., Zhao, X., Shi, X., Teng, H., Zhong, J., You, M., Xia, K., Sun, Z., and Mao, F. (2021). OncoVar: an integrated database and analysis platform for oncogenic driver variants in cancers. Nucleic acids research 49, D1289-d1301. 10.1093/nar/gkaa1033.

Yang, J., Xu, J., Wang, W., Zhang, B., Yu, X., and Shi, S. (2023). Epigenetic regulation in the tumor microenvironment: molecular mechanisms and therapeutic targets. Signal Transduction and Targeted Therapy 8, 210. 10.1038/s41392-023-01480-x.

Lv, Y., Lv, Y., Wang, Z., Yuan, K., and Zeng, Y. (2022). Noncoding RNAs as sensors of tumor microenvironmental stress. Journal of Experimental & Clinical Cancer Research 41, 224. 10.1186/s13046-022-02433-y.

*4.      In general, figures need to be reworked. Legends need to include more details regarding what statistics are reported along the figures and how to interpret them. Figure arrangement, panel and font size consistency, and readability need to be improved.*

**Response:** Thanks for the comments. We have imworked figures as suggested by the reviewer.

*Minor comments*

*5.      The order of the figure panels should match the text. Specifically, figure 1b & c are LUAD validation and should go with figure 4c when the single-cell studies are talked about in the results section.*

**Response:** Thanks for the suggestion. We have rearranged the Figures to match the text accordingly. The initial Figures 1b and 1c have moved to Figure 4.

*6.      The immune traits were transformed to quantitative or binary values depending on their distributions, which are categorized into three distinct patterns. More reasonings are needed here, e.g., what does raw trait of 0 mean in different situations? Are they specific to a subgroup of immune traits or cancer type?*

**Response:** We apologize for the brevity of our discussion regarding trait transformation. The basic idea of the transformation follows previous studies of Sayaman et al, in which parts of the TME traits were also dichotomized and treated as binary variables in case of a large number of zero values. For example, for ~ 50% of the participants, the corresponding TME traits of B Cells Memory (%) are 0. In this case, the trait cannot be normalized, as requested by linear regression. The data transformation process is completely data-driven and remains the same for all TME traits and cancer types. The above discussion has been added in the revised manuscript **(lines 389-394)**.

**Reference:**	Sayaman, R.W., Saad, M., Thorsson, V., Hu, D., Hendrickx, W., Roelands, J., Porta-Pardo, E., Mokrab, Y., Farshidfar, F., and Kirchhoff, T. (2021). Germline genetic contribution to the immune landscape of cancer. Immunity 54, 367-386.

*7.      In figure 3a, would like to see other hits as well to show that KEAP1 is the ‘top gene’.*

**Response**: We have added other hits in Figure 3a. We define top genes as the genes whose FDR q-values < 0.05. To avoid the misunderstanding, we replaced the phrase ‘top gene’ using ‘gene shaping multiple LUAD TMEs in LUAD’ in the Figure 3a (see line 721).

*8.      In figure 4b, show statistical test comparing NRF2 signature scores of KEAP1 WT and mutated samples.*

**Response:** Thanks to the reviewer’s comments, we performed one-sided Wilcoxon tests to compare NRF2 signature score distribution between TCGA samples with or without KEAP1 mutation and got significant pvalue score < 2.2e-16 (alternative hypothesis: NRF2sigScoreKEAP1_mutated greater than NRF2sigScoreKEAP1_wt) which suggests that samples with KEAP1 mutations are having significantly higher NFR2 signature score than samples without KEAP1 mutation. We also added the pvalue to figure 4b.

*9.      Figure 5b panels and legends do not match. Bottom panel of figure 5a unclear, e.g. what are the lines and boxes, sizes of the box mean, etc. It is not clear what normalized expression is plotted in figure 5e heatmap and how it addresses the claims made in the text. Should it be correlation heatmap instead?*

**Response:** Thanks to the reviewer’s comments. We have corrected Figure 5b legends and added more description in Figure 5a and Figure 5e. In Figure 5e, color in the heatmap represent the normalized expression of each ligand. Since we are more interested in the association within malignant cells, we specially aggregated the malignant cells of each tumor samples into pseudobulk data and calculate the averaged gene expression for each ligand as well as NRF2 gene signature. By performing correlation analysis, we were able to identify top ligands whose expression are highly associated with NRF2 activity. In Figure 5e, row represents ligands that are ranked by its correlation with NRF2 signature (top to bottom : highest to lowest). Column represents tumor samples that are ranked by NRF2 gene signature(left to right : highest to lowest). Colors in the heatmap represent the averaged expression level of each ligand in pseudo bulk data that are normalized across different samples.

*10.      FAM3C stratified patient groups in figure 6c do not show statistical differences.*

**Response:** Thanks for your comment. We have rephased the statement in manuscript as following:

**(lines 287-293)**	“For other inhibitory ligands whose expression is highly associated with NRF2 signature, we found that PVR expression is associated with worse survival in LUAD patient cohort significantly (Figure 6d, p.value < 0.01). Expression of FAM3C also shows trend of association with worse survival in LUAD patients after 50 months although the p value is not significant due to the fact that only small number of patients live longer than 50 months (Figure 6c, p.value = 0.16). We report these findings to the field to support new immunotherapy target identification for treating these NRF2 pathway activated LUAD patients.”

*11.      The x-axis timelines for survival plots in figure 6c, d and figure 7b seem suspiciously long (250 months). Please double check the analysis and plotting.*

**Response:** Most of individuals are censored less than 100 months but we do observe a few individuals with a long follow-up record. The examples include sample of TCGA-49-AARQ censored at around 224 months, sample of TCGA-78-7163 censored at around 242 months, and sample of TCGA-78-8640 censored at around 235 months. **(lines 759-761)**

Figure S1. Sensitivity analyses results of the significant associations between genes and TME traits. In all the four panels, the x-axis corresponds to the analysis including only coding regions (denoted as Coding). The y-axis corresponds to the alternative analyses as below. (a) the somatic mutations in non-coding regions were additionally included (denoted as NonCoding); (b) driver somatic mutations were double-weighted (denoted as Driver); (c) only somatic mutations classified as Nonsense Mutation were included (denoted as Nonsense); (d) only somatic mutations classified as Missense Mutation were included (denoted as Missense). If a gene was identified as significant in both Coding and the alternative analyses, the gene was denoted as “Both” whose x-axis and y-axis are for the corresponding p values. If the gene was only identified in Coding analyses, the gene was denoted as “Coding” whose x-axis and y-axis are for the p values in the Coding analyses. If the gene was only identified in the alternative analyses, the gene was denoted as the alternative analysis whose x-axis and y-axis are for the p values in the alternative analyses.
