# Supplementary material for: Genome-wide analyses reveal the contribution of somatic variants to the immune landscape of multiple cancer types
Source: PLoS Genet. 2024 Jan 19;20(1):e1011134. doi: 10.1371/journal.pgen.1011134 (PMC10829993; doi:10.1371/journal.pgen.1011134)
Supplement: Attachment — Submitted filename: Response_to_Reviewers_2024-01-05.docx [file pgen.1011134.s013.docx]

We would like to thank the reviewer for the careful examination of the manuscript. Please find below the comment from the reviewer (in italics) and our responses after each comment.

**Reviewer #2:** *The authors addressed most of the comments very well.*

**We would like to thank you for the positive comment.**

*1.      Minor comment: In the additional sensitivity analysis figure S1, the figure contents are somewhat confusing. The x, y-axes labels are supposed to be statistical significance from Coding and alternative analyses, respectively. However, for the genes that were only identified in one of the Coding or alternative analyses, the x,y values of the genes are the same (the significant p-value), making the points always fall on the x=y line, and therefore the xy axis definitions are not aligned. I found this design misleading. Is there a specific reason against plotting the p-values as they are? In addition, can you make some general comments on the characteristics (if any) of the genes identified only in the alternative analyses and why they are not of interest.*

**Response:** Thank you for your comment. In our analysis, genes with fewer than 5 somatic mutation carriers were excluded for both coding and alternative analyses. Consequently, the sets of genes included in different analyses were not identical. We agree that the design could potentially lead to a misunderstanding, and we have updated Figure S1 to address this concern.

In the revised Figure S1, for genes included in both coding and alternative analyses, we display p-values irrespective of their significance. Meanwhile, for genes not included in either coding or alternative analyses, we set the corresponding p-value to 1. We believe that these adjustments enhance the clarity and accuracy of our presentation.

We checked the genes exclusively identified by alternative analyses and revised the statements in the Discussion Section as below.

“Incorporating the somatic mutations in non-coding regions revealed a slightly broader spectrum of findings, of which potentially significant discoveries include UBTF in Ovarian Cancer (OV) and ACTBL2 in HNSC (Table S6).”
